# Poly[bis­(4,4′-bipyridine)(μ_3_-4,4′-dicarboxybiphenyl-3,3′-di­carboxyl­ato)iron(II)]

**DOI:** 10.1107/S1600536809046273

**Published:** 2009-11-07

**Authors:** Qun-Hui Meng, Hui-Ling Lai, Han Lu, Yi-Fan Luo, Rong-Hua Zeng

**Affiliations:** a School of Chemistry and Environment, South China Normal University, Guangzhou 510006, People’s Republic of China; b South China Normal University, Key Laboratory of Technology of Electrochemical Energy Storage and Power Generation, in Guangdong Universities, Guangzhou 510006, People’s Republic of China

## Abstract

In the polymeric title complex, [Fe(C_16_H_8_O_8_)(C_10_H_8_N_2_)_2_]_*n*_, the iron(II) cation is coordinated by four O atoms from three different 4,4′-dicarboxybiphenyl-3,3′-di­carboxyl­ate ligands and two N atoms from two 4,4′-bipyridine ligands in a distorted octa­hedral geometry. The 4,4′-dicarboxybiphenyl-3,3′-di­carboxyl­ate ligands bridge adjacent cations, forming chains parallel to the *c* axis. The chains are further connected by inter­molecular O—H⋯N hydrogen bonds, forming two-dimensional supra­molecular layers parallel to (010).

## Related literature

For general background to self-assembling coordination polymers, see: Li *et al.* (2008 ▶); Yaghi *et al.* (2003 ▶). For related structures, see: Li *et al.* (2009 ▶); Liu *et al.* (2009 ▶); Wang *et al.* (2007 ▶).
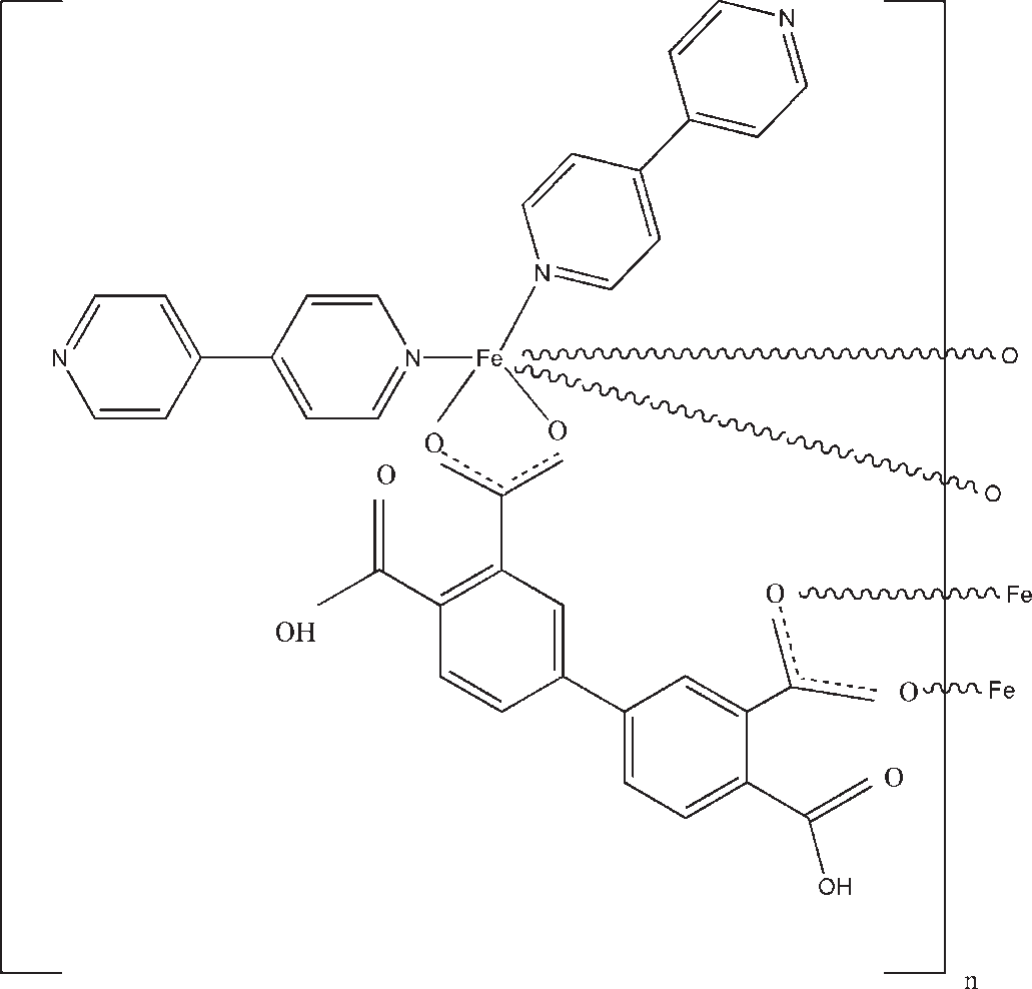


## Experimental

### 

#### Crystal data

[Fe(C_16_H_8_O_8_)(C_10_H_8_N_2_)_2_]
*M* *_r_* = 696.44
Monoclinic, 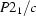

*a* = 11.9569 (13) Å
*b* = 24.114 (3) Å
*c* = 10.7232 (12) Å
β = 105.855 (1)°
*V* = 2974.2 (6) Å^3^
*Z* = 4
Mo *K*α radiation
μ = 0.57 mm^−1^
*T* = 296 K
0.23 × 0.21 × 0.19 mm

#### Data collection

Bruker APEXII area-detector diffractometer
Absorption correction: multi-scan (*SADABS*; Bruker, 2005 ▶) *T*_min_ = 0.880, *T*_max_ = 0.899
15189 measured reflections
5363 independent reflections
4293 reflections with *I* > 2σ(*I*)
*R*_int_ = 0.038

#### Refinement

*R*[*F* ^2^ > 2σ(*F* ^2^)] = 0.037
*wR*(*F* ^2^) = 0.100
*S* = 1.03
5363 reflections
444 parameters
H-atom parameters constrained
Δρ_max_ = 0.71 e Å^−3^
Δρ_min_ = −0.46 e Å^−3^

### 

Data collection: *APEX2* (Bruker, 2005 ▶); cell refinement: *SAINT* (Bruker, 2005 ▶); data reduction: *SAINT*; program(s) used to solve structure: *SHELXS97* (Sheldrick, 2008 ▶); program(s) used to refine structure: *SHELXL97* (Sheldrick, 2008 ▶); molecular graphics: *SHELXTL* (Sheldrick, 2008 ▶); software used to prepare material for publication: *SHELXTL*.

## Supplementary Material

Crystal structure: contains datablocks I, global. DOI: 10.1107/S1600536809046273/rz2381sup1.cif

Structure factors: contains datablocks I. DOI: 10.1107/S1600536809046273/rz2381Isup2.hkl

Additional supplementary materials:  crystallographic information; 3D view; checkCIF report

## Figures and Tables

**Table 1:** Hydrogen-bond geometry (Å, °)

*D*—H⋯*A*	*D*—H	H⋯*A*	*D*⋯*A*	*D*—H⋯*A*
O5—H5⋯N2^i^	0.82	1.86	2.677 (2)	171
O3—H3⋯N4^ii^	0.82	1.81	2.598 (3)	162

Symmetry codes: (i) 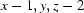; (ii) 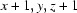.

**Fig. 1.**
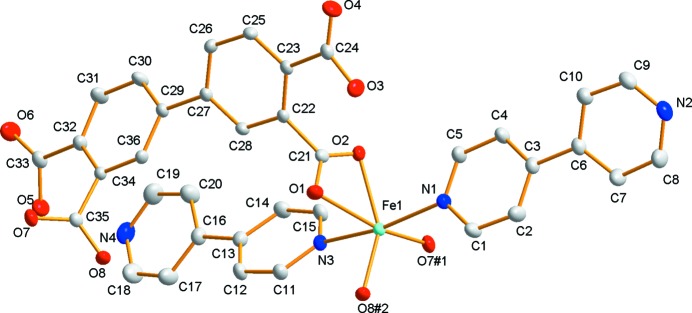
The molecular structure of the title compound showing the atomic-numbering scheme. Displacement ellipsoids drawn at the 30% probability level. H atoms are omitted for clarity. [Symmetry codes: (#1) x, y, 1+z; (#2) 1-x, 2-y, 1-z]

**Fig. 2.**
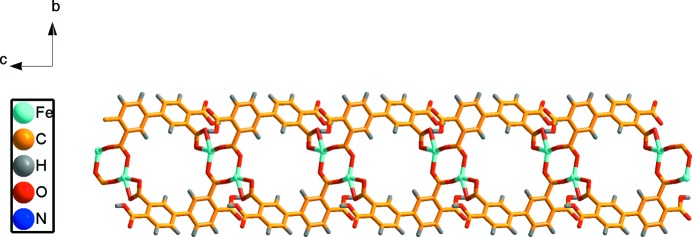
Partial crystal packing of the title compound showing a one-dimensional chain running parallel to the c axis. 4,4'-Bipyridine ligands are omitted for clarity.

**Fig. 3.**
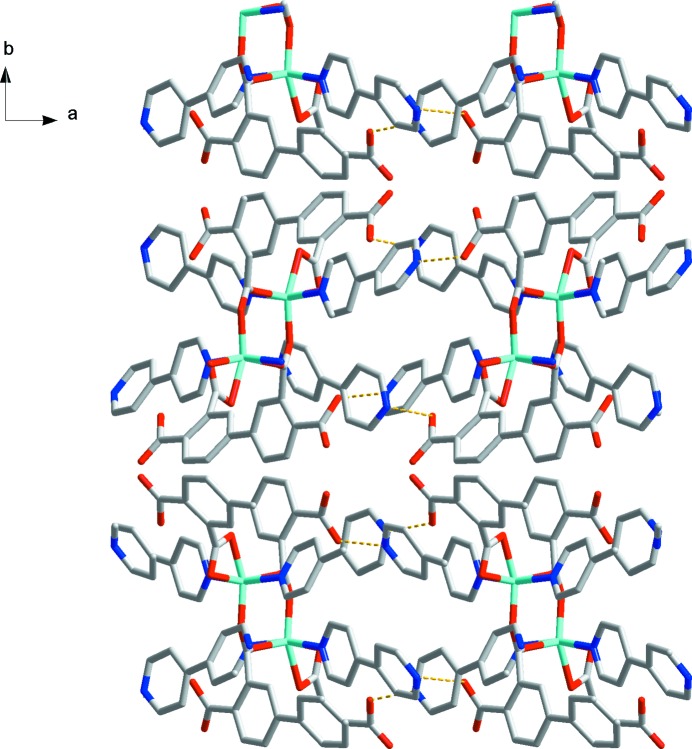
Partial crystal packing of the title compound showing the two-dimensional supramolecular layers parallel to (0 1 0). Hydrogen bonds are shown as dashed lines.

## supplementary crystallographic information

### Comment

The construction of self-assembling coordination polymers is of current interest in the fields of supramolecular chemistry and crystal engineering, because of their potential applications in gas storage, molecular sieves, ion exchange, catalysis, magnetism, nonlinearoptics, and molecular sensing (Li *et al.*, 2008; Yaghi *et al.*, 2003). Due to the presence of four carboxylic groups, 4,4'-dicarboxybiphenyl-3,3'-dicarboxylate ligands are promising building blocks for the construction of novel metal-organic coordination polymers (Li *et al.*, 2009; Liu *et al.*, 2009; Wang *et al.*, 2007). Herein, we report the title new metal-organic framework, which was synthesized under hydrothermal conditions.

In the title complex (Fig. 1), each iron(II) atom exhibits a distorted octahedral geometry, defined by four oxygen atoms from three different 4,4'-dicarboxybiphenyl-3,3'-dicarboxylate ligands and two nitrogen atoms from two different 4,4'-bipyridine ligands. The Fe—O and Fe—N distances range from 2.0244 (1) to 2.3327 (1) Å and 2.2201 (1) to 2.2615 (1) Å, respectively, while the O—Fe—O angles and N—Fe—O angles fall in the range from 58.64 (6) to 160.43 (7) %A and 82.29 (6) to 102.91 (7) %A, respectively. The dihedral angles between the N1/C1–C5 and N2/C6–C10, N3/C11–C15 and N4/C16–C20, C22/C23/C25–c28 and C29–C32/C33/C34 are 25.74 (8), 26.91 (9) and 37.39 (7)°, respectively. Adjacent metal centres are connected by the 4,4'-dicarboxybiphenyl-3,3'-dicarboxylate ligands to form chains (Fig. 2) running parallel to the *c* axis. Intermolecular O—H···N hydrogen bonds (Table 1) link the chains into two-dimensional supramolecular layers parallel to (0 1 0) (Fig. 3).

### Experimental

A mixture of FeSO_4_.7H_2_O (0.139 g, 0.5mmol), 4,4'-bipyridine (0.078 g; 0.5 mmol), biphenyl-3,3',-4,4'-tetracarboxylic acid (0.165 g; 0.5 mmol), water (10mL) were stirred vigorously for 30 min and then sealed in a Teflon-lined stainless-steel autoclave (25 mL capacity). The autoclave was heated and maintained at 423K for 3 days, then cooled to room temperature at 5Kh^-1^. Red block crystals suitable for X-ray analysis were obtained.

### Refinement

Carboxy H atoms were located in a difference Fourier map and refined using a riding model approximation, with O–H = 0.82 Å and with 1.5 *U*_eq_(O). All other H atoms were placed at calculated positions and treated as riding on parent atoms with C—H = 0.93 Å, and with *U*_iso_(H) = 1.2 *U*_eq_(C).

### Crystal data

**Table:**

[Fe(C_16_H_8_O_8_)(C_10_H_8_N_2_)_2_]	*F*(000) = 1432
*M**_r_* = 696.44	*D*_x_ = 1.555 Mg m^−^^3^
Monoclinic, *P*2_1_/*c*	Mo *K*α radiation, λ = 0.71073 Å
Hall symbol: -P 2ybc	Cell parameters from 4905 reflections
*a* = 11.9569 (13) Å	θ = 2.4–27.0°
*b* = 24.114 (3) Å	µ = 0.57 mm^−^^1^
*c* = 10.7232 (12) Å	*T* = 296 K
β = 105.855 (1)°	Block, red
*V* = 2974.2 (6) Å^3^	0.23 × 0.21 × 0.19 mm
*Z* = 4	

### Data collection

**Table:**

Bruker APEXII area-detector diffractometer	5363 independent reflections
Radiation source: fine-focus sealed tube	4293 reflections with *I* > 2σ(*I*)
graphite	*R*_int_ = 0.038
φ and ω scans	θ_max_ = 25.2°, θ_min_ = 1.7°
Absorption correction: multi-scan (*SADABS*; Bruker, 2005)	*h* = −14→10
*T*_min_ = 0.880, *T*_max_ = 0.899	*k* = −28→26
15189 measured reflections	*l* = −12→12

### Refinement

**Table:**

Refinement on *F*^2^	Primary atom site location: structure-invariant direct methods
Least-squares matrix: full	Secondary atom site location: difference Fourier map
*R*[*F*^2^ > 2σ(*F*^2^)] = 0.037	Hydrogen site location: inferred from neighbouring sites
*wR*(*F*^2^) = 0.100	H-atom parameters constrained
*S* = 1.03	*w* = 1/[σ^2^(*F*_o_^2^) + (0.0462*P*)^2^ + 1.5067*P*] where *P* = (*F*_o_^2^ + 2*F*_c_^2^)/3
5363 reflections	(Δ/σ)_max_ = 0.002
444 parameters	Δρ_max_ = 0.71 e Å^−^^3^
0 restraints	Δρ_min_ = −0.46 e Å^−^^3^

### Special details

**Table:**

Geometry. All esds (except the esd in the dihedral angle between two l.s. planes) are estimated using the full covariance matrix. The cell esds are taken into account individually in the estimation of esds in distances, angles and torsion angles; correlations between esds in cell parameters are only used when they are defined by crystal symmetry. An approximate (isotropic) treatment of cell esds is used for estimating esds involving l.s. planes.
Refinement. Refinement of F^2^ against ALL reflections. The weighted R-factor wR and goodness of fit S are based on F^2^, conventional R-factors R are based on F, with F set to zero for negative F^2^. The threshold expression of F^2^ > 2sigma(F^2^) is used only for calculating R-factors(gt) etc. and is not relevant to the choice of reflections for refinement. R-factors based on F^2^ are statistically about twice as large as those based on F, and R- factors based on ALL data will be even larger.

### Fractional atomic coordinates and isotropic or equivalent isotropic displacement parameters (Å^2^)

**Table:**

	*x*	*y*	*z*	*U*_iso_*/*U*_eq_	
Fe1	0.58299 (3)	0.948957 (13)	0.88381 (3)	0.02617 (11)	
C27	0.63026 (19)	0.80826 (9)	0.4436 (2)	0.0267 (5)	
C11	0.4184 (2)	0.97360 (10)	0.5952 (2)	0.0331 (6)	
H11	0.4542	1.0082	0.6064	0.040*	
O3	0.88485 (15)	0.84329 (7)	0.88903 (16)	0.0417 (4)	
H3	0.9409	0.8416	0.9532	0.063*	
C29	0.5469 (2)	0.81316 (9)	0.3128 (2)	0.0277 (5)	
N2	1.05946 (18)	0.88387 (10)	1.71537 (19)	0.0416 (5)	
C3	0.8429 (2)	0.91461 (10)	1.3316 (2)	0.0322 (5)	
O5	0.23281 (15)	0.87426 (7)	−0.06664 (16)	0.0414 (4)	
H5	0.1792	0.8734	−0.1333	0.062*	
C21	0.6846 (2)	0.88567 (9)	0.7580 (2)	0.0279 (5)	
N1	0.70900 (17)	0.93693 (8)	1.07668 (18)	0.0327 (5)	
N4	0.0513 (2)	0.85951 (11)	0.0994 (2)	0.0510 (6)	
N3	0.44003 (17)	0.93946 (8)	0.69706 (18)	0.0304 (4)	
C10	0.9865 (2)	0.85667 (11)	1.4936 (2)	0.0382 (6)	
H10	0.9875	0.8309	1.4293	0.046*	
C5	0.7413 (2)	0.88541 (11)	1.1179 (2)	0.0388 (6)	
H5A	0.7179	0.8564	1.0593	0.047*	
C2	0.8088 (2)	0.96780 (11)	1.2899 (2)	0.0435 (7)	
H2	0.8296	0.9974	1.3471	0.052*	
C6	0.9155 (2)	0.90314 (11)	1.4651 (2)	0.0332 (5)	
C1	0.7440 (2)	0.97724 (11)	1.1640 (2)	0.0432 (7)	
H1	0.7237	1.0136	1.1386	0.052*	
C4	0.8068 (2)	0.87257 (11)	1.2414 (2)	0.0383 (6)	
H4	0.8266	0.8359	1.2642	0.046*	
C9	1.0557 (2)	0.84914 (12)	1.6183 (2)	0.0414 (6)	
H9	1.1026	0.8177	1.6355	0.050*	
C7	0.9158 (2)	0.93795 (12)	1.5675 (3)	0.0482 (7)	
H7	0.8675	0.9689	1.5544	0.058*	
C8	0.9880 (3)	0.92674 (13)	1.6896 (2)	0.0506 (7)	
H8	0.9861	0.9506	1.7571	0.061*	
C12	0.3463 (2)	0.96087 (10)	0.4744 (2)	0.0338 (6)	
H12	0.3344	0.9864	0.4069	0.041*	
C16	0.2130 (2)	0.89236 (11)	0.3283 (2)	0.0350 (6)	
C14	0.3124 (2)	0.87458 (10)	0.5620 (2)	0.0358 (6)	
H14	0.2764	0.8401	0.5547	0.043*	
C17	0.1517 (3)	0.93092 (12)	0.2401 (3)	0.0519 (8)	
H17	0.1640	0.9687	0.2559	0.062*	
C13	0.2917 (2)	0.90935 (10)	0.4548 (2)	0.0313 (5)	
C15	0.3853 (2)	0.89073 (10)	0.6785 (2)	0.0345 (6)	
H15	0.3973	0.8664	0.7482	0.041*	
C20	0.1939 (2)	0.83734 (12)	0.2949 (3)	0.0484 (7)	
H20	0.2353	0.8100	0.3495	0.058*	
C19	0.1136 (3)	0.82278 (13)	0.1808 (3)	0.0527 (8)	
H19	0.1027	0.7854	0.1601	0.063*	
C18	0.0727 (3)	0.91279 (14)	0.1290 (3)	0.0622 (9)	
H18	0.0318	0.9392	0.0712	0.075*	
C26	0.7131 (2)	0.76653 (10)	0.4723 (2)	0.0313 (5)	
H26	0.7138	0.7393	0.4112	0.038*	
C36	0.51638 (19)	0.86533 (9)	0.2589 (2)	0.0269 (5)	
H36	0.5478	0.8966	0.3066	0.032*	
C22	0.70735 (19)	0.84500 (9)	0.66113 (19)	0.0246 (5)	
C25	0.7947 (2)	0.76543 (10)	0.5919 (2)	0.0305 (5)	
H25	0.8516	0.7380	0.6087	0.037*	
C34	0.44068 (19)	0.87230 (9)	0.1366 (2)	0.0250 (5)	
C23	0.79408 (19)	0.80409 (9)	0.6875 (2)	0.0258 (5)	
C33	0.2957 (2)	0.82910 (10)	−0.0585 (2)	0.0344 (6)	
C28	0.62884 (19)	0.84684 (9)	0.5393 (2)	0.0284 (5)	
H28	0.5733	0.8749	0.5210	0.034*	
C30	0.5005 (2)	0.76689 (10)	0.2393 (2)	0.0394 (6)	
H30	0.5216	0.7315	0.2717	0.047*	
C24	0.8873 (2)	0.80139 (10)	0.8127 (2)	0.0296 (5)	
C32	0.3894 (2)	0.82577 (10)	0.0668 (2)	0.0310 (5)	
C31	0.4231 (2)	0.77351 (10)	0.1183 (2)	0.0415 (7)	
H31	0.3929	0.7422	0.0700	0.050*	
O2	0.62783 (14)	0.86750 (7)	0.83378 (14)	0.0317 (4)	
O1	0.70685 (16)	0.93579 (7)	0.75185 (16)	0.0382 (4)	
O4	0.95713 (16)	0.76380 (8)	0.83860 (17)	0.0479 (5)	
O6	0.27834 (18)	0.79341 (8)	−0.14063 (18)	0.0575 (6)	
C35	0.43099 (19)	0.92924 (9)	0.0796 (2)	0.0256 (5)	
O7	0.45273 (14)	0.93505 (7)	−0.02766 (14)	0.0338 (4)	
O8	0.40781 (15)	0.96848 (7)	0.14606 (15)	0.0355 (4)	

### Atomic displacement parameters (Å^2^)

**Table:**

	*U*^11^	*U*^22^	*U*^33^	*U*^12^	*U*^13^	*U*^23^
Fe1	0.0328 (2)	0.02408 (19)	0.01910 (18)	0.00295 (13)	0.00281 (13)	−0.00112 (12)
C27	0.0317 (13)	0.0253 (12)	0.0203 (11)	0.0002 (9)	0.0025 (10)	0.0004 (9)
C11	0.0381 (14)	0.0307 (13)	0.0265 (12)	−0.0020 (10)	0.0023 (10)	−0.0001 (10)
O3	0.0371 (11)	0.0483 (11)	0.0302 (9)	0.0044 (8)	−0.0070 (7)	−0.0103 (8)
C29	0.0330 (13)	0.0271 (12)	0.0209 (11)	0.0031 (10)	0.0036 (10)	0.0012 (9)
N2	0.0345 (12)	0.0527 (14)	0.0309 (12)	−0.0021 (10)	−0.0023 (9)	0.0060 (10)
C3	0.0269 (13)	0.0415 (15)	0.0247 (12)	0.0026 (10)	0.0010 (10)	−0.0004 (10)
O5	0.0370 (11)	0.0463 (11)	0.0307 (10)	0.0071 (8)	−0.0083 (8)	−0.0022 (8)
C21	0.0298 (13)	0.0275 (13)	0.0208 (11)	0.0054 (10)	−0.0024 (9)	−0.0008 (9)
N1	0.0348 (12)	0.0340 (12)	0.0251 (10)	0.0045 (9)	0.0012 (9)	−0.0007 (8)
N4	0.0408 (14)	0.0683 (18)	0.0352 (13)	−0.0011 (12)	−0.0041 (10)	−0.0144 (12)
N3	0.0311 (11)	0.0349 (12)	0.0235 (10)	0.0017 (8)	0.0047 (8)	−0.0001 (8)
C10	0.0369 (15)	0.0401 (15)	0.0328 (13)	0.0020 (11)	0.0014 (11)	0.0013 (11)
C5	0.0485 (16)	0.0371 (14)	0.0241 (12)	0.0025 (12)	−0.0013 (11)	−0.0029 (10)
C2	0.0490 (17)	0.0376 (15)	0.0335 (14)	0.0063 (12)	−0.0065 (12)	−0.0082 (11)
C6	0.0270 (13)	0.0425 (15)	0.0263 (12)	−0.0008 (10)	0.0008 (10)	0.0018 (10)
C1	0.0532 (17)	0.0352 (15)	0.0307 (14)	0.0117 (12)	−0.0062 (12)	0.0001 (11)
C4	0.0473 (16)	0.0349 (14)	0.0259 (12)	0.0050 (11)	−0.0013 (11)	0.0028 (10)
C9	0.0344 (15)	0.0457 (16)	0.0385 (15)	0.0047 (12)	0.0004 (11)	0.0098 (12)
C7	0.0495 (18)	0.0548 (18)	0.0308 (14)	0.0195 (13)	−0.0051 (12)	−0.0021 (12)
C8	0.0538 (18)	0.0607 (19)	0.0289 (14)	0.0114 (15)	−0.0031 (12)	−0.0048 (13)
C12	0.0372 (14)	0.0360 (14)	0.0239 (12)	−0.0020 (11)	0.0013 (10)	0.0044 (10)
C16	0.0298 (13)	0.0428 (15)	0.0279 (12)	−0.0010 (11)	0.0004 (10)	−0.0044 (11)
C14	0.0337 (14)	0.0323 (14)	0.0370 (14)	−0.0048 (10)	0.0020 (11)	0.0009 (11)
C17	0.0584 (19)	0.0431 (17)	0.0396 (16)	0.0095 (14)	−0.0115 (13)	−0.0097 (13)
C13	0.0262 (13)	0.0370 (14)	0.0273 (12)	0.0026 (10)	0.0015 (10)	−0.0040 (10)
C15	0.0354 (14)	0.0371 (14)	0.0283 (12)	−0.0022 (11)	0.0042 (10)	0.0060 (10)
C20	0.0502 (17)	0.0414 (16)	0.0422 (16)	−0.0034 (13)	−0.0067 (13)	−0.0029 (12)
C19	0.0542 (19)	0.0511 (18)	0.0448 (16)	−0.0116 (14)	0.0000 (14)	−0.0133 (14)
C18	0.062 (2)	0.065 (2)	0.0414 (17)	0.0152 (16)	−0.0162 (15)	−0.0066 (15)
C26	0.0390 (14)	0.0292 (13)	0.0233 (11)	0.0060 (10)	0.0048 (10)	−0.0049 (9)
C36	0.0336 (13)	0.0241 (12)	0.0200 (11)	0.0001 (9)	0.0022 (9)	−0.0040 (9)
C22	0.0300 (12)	0.0233 (12)	0.0196 (11)	−0.0002 (9)	0.0050 (9)	0.0009 (8)
C25	0.0345 (13)	0.0278 (12)	0.0271 (12)	0.0094 (10)	0.0048 (10)	0.0008 (9)
C34	0.0285 (12)	0.0258 (12)	0.0188 (11)	0.0029 (9)	0.0034 (9)	−0.0006 (9)
C23	0.0272 (12)	0.0263 (12)	0.0220 (11)	−0.0011 (9)	0.0036 (9)	0.0024 (9)
C33	0.0363 (14)	0.0338 (14)	0.0274 (12)	−0.0049 (11)	−0.0011 (10)	0.0018 (10)
C28	0.0304 (13)	0.0272 (12)	0.0247 (11)	0.0076 (9)	0.0027 (10)	0.0015 (9)
C30	0.0529 (17)	0.0250 (13)	0.0306 (13)	0.0028 (11)	−0.0049 (11)	0.0022 (10)
C24	0.0286 (13)	0.0348 (14)	0.0235 (11)	−0.0014 (10)	0.0039 (10)	0.0030 (10)
C32	0.0354 (13)	0.0296 (13)	0.0237 (11)	−0.0011 (10)	0.0011 (10)	−0.0013 (10)
C31	0.0551 (18)	0.0253 (13)	0.0331 (14)	−0.0048 (11)	−0.0068 (12)	−0.0038 (10)
O2	0.0383 (10)	0.0346 (9)	0.0204 (8)	0.0044 (7)	0.0049 (7)	−0.0013 (7)
O1	0.0517 (11)	0.0256 (9)	0.0346 (9)	0.0016 (8)	0.0076 (8)	−0.0032 (7)
O4	0.0446 (11)	0.0457 (12)	0.0422 (11)	0.0147 (9)	−0.0071 (8)	0.0026 (9)
O6	0.0701 (14)	0.0466 (12)	0.0369 (11)	0.0030 (10)	−0.0176 (9)	−0.0143 (9)
C35	0.0243 (12)	0.0267 (12)	0.0210 (11)	−0.0005 (9)	−0.0017 (9)	−0.0005 (9)
O7	0.0377 (10)	0.0395 (10)	0.0233 (8)	0.0002 (7)	0.0068 (7)	0.0056 (7)
O8	0.0532 (11)	0.0234 (9)	0.0284 (9)	0.0038 (7)	0.0084 (8)	−0.0023 (7)

### Geometric parameters (Å, °)

**Table:**

Fe1—O8^i^	2.0244 (17)	C7—C8	1.384 (4)
Fe1—O7^ii^	2.0609 (16)	C7—H7	0.9300
Fe1—O2	2.1426 (16)	C8—H8	0.9300
Fe1—N1	2.2201 (19)	C12—C13	1.392 (3)
Fe1—N3	2.2615 (19)	C12—H12	0.9300
Fe1—O1	2.3327 (18)	C16—C20	1.377 (4)
C27—C26	1.387 (3)	C16—C17	1.384 (4)
C27—C28	1.389 (3)	C16—C13	1.482 (3)
C27—C29	1.487 (3)	C14—C15	1.370 (3)
C11—N3	1.335 (3)	C14—C13	1.390 (3)
C11—C12	1.381 (3)	C14—H14	0.9300
C11—H11	0.9300	C17—C18	1.374 (4)
O3—C24	1.306 (3)	C17—H17	0.9300
O3—H3	0.8200	C15—H15	0.9300
C29—C36	1.391 (3)	C20—C19	1.379 (4)
C29—C30	1.391 (3)	C20—H20	0.9300
N2—C8	1.321 (4)	C19—H19	0.9300
N2—C9	1.327 (3)	C18—H18	0.9300
C3—C2	1.383 (4)	C26—C25	1.383 (3)
C3—C4	1.386 (3)	C26—H26	0.9300
C3—C6	1.483 (3)	C36—C34	1.385 (3)
O5—C33	1.313 (3)	C36—H36	0.9300
O5—H5	0.8200	C22—C28	1.386 (3)
C21—O1	1.243 (3)	C22—C23	1.403 (3)
C21—O2	1.271 (3)	C25—C23	1.387 (3)
C21—C22	1.506 (3)	C25—H25	0.9300
N1—C1	1.335 (3)	C34—C32	1.395 (3)
N1—C5	1.339 (3)	C34—C35	1.495 (3)
N4—C19	1.321 (4)	C23—C24	1.494 (3)
N4—C18	1.331 (4)	C33—O6	1.208 (3)
N3—C15	1.333 (3)	C33—C32	1.498 (3)
C10—C9	1.379 (3)	C28—H28	0.9300
C10—C6	1.389 (3)	C30—C31	1.382 (3)
C10—H10	0.9300	C30—H30	0.9300
C5—C4	1.378 (3)	C24—O4	1.212 (3)
C5—H5A	0.9300	C32—C31	1.391 (3)
C2—C1	1.380 (3)	C31—H31	0.9300
C2—H2	0.9300	C35—O7	1.254 (3)
C6—C7	1.382 (4)	C35—O8	1.260 (3)
C1—H1	0.9300	O7—Fe1^iii^	2.0609 (16)
C4—H4	0.9300	O8—Fe1^i^	2.0244 (17)
C9—H9	0.9300		
			
O8^i^—Fe1—O7^ii^	108.27 (7)	C13—C12—H12	120.4
O8^i^—Fe1—O2	146.50 (7)	C20—C16—C17	116.8 (2)
O7^ii^—Fe1—O2	103.80 (7)	C20—C16—C13	121.5 (2)
O8^i^—Fe1—N1	102.91 (7)	C17—C16—C13	121.6 (2)
O7^ii^—Fe1—N1	87.46 (7)	C15—C14—C13	120.5 (2)
O2—Fe1—N1	87.76 (7)	C15—C14—H14	119.7
O8^i^—Fe1—N3	91.15 (7)	C13—C14—H14	119.7
O7^ii^—Fe1—N3	84.72 (7)	C18—C17—C16	119.2 (3)
O2—Fe1—N3	82.29 (6)	C18—C17—H17	120.4
N1—Fe1—N3	165.49 (8)	C16—C17—H17	120.4
O8^i^—Fe1—O1	88.10 (6)	C14—C13—C12	116.3 (2)
O7^ii^—Fe1—O1	160.43 (7)	C14—C13—C16	121.0 (2)
O2—Fe1—O1	58.64 (6)	C12—C13—C16	122.6 (2)
N1—Fe1—O1	99.53 (7)	N3—C15—C14	123.4 (2)
N3—Fe1—O1	84.29 (7)	N3—C15—H15	118.3
C26—C27—C28	118.3 (2)	C14—C15—H15	118.3
C26—C27—C29	121.45 (19)	C16—C20—C19	120.1 (3)
C28—C27—C29	120.2 (2)	C16—C20—H20	120.0
N3—C11—C12	124.2 (2)	C19—C20—H20	120.0
N3—C11—H11	117.9	N4—C19—C20	123.1 (3)
C12—C11—H11	117.9	N4—C19—H19	118.5
C24—O3—H3	109.5	C20—C19—H19	118.5
C36—C29—C30	118.1 (2)	N4—C18—C17	123.7 (3)
C36—C29—C27	119.7 (2)	N4—C18—H18	118.1
C30—C29—C27	122.1 (2)	C17—C18—H18	118.1
C8—N2—C9	116.6 (2)	C25—C26—C27	120.0 (2)
C2—C3—C4	116.4 (2)	C25—C26—H26	120.0
C2—C3—C6	121.9 (2)	C27—C26—H26	120.0
C4—C3—C6	121.7 (2)	C34—C36—C29	122.2 (2)
C33—O5—H5	109.5	C34—C36—H36	118.9
O1—C21—O2	121.9 (2)	C29—C36—H36	118.9
O1—C21—C22	121.0 (2)	C28—C22—C23	119.0 (2)
O2—C21—C22	116.4 (2)	C28—C22—C21	115.20 (19)
C1—N1—C5	115.9 (2)	C23—C22—C21	125.58 (19)
C1—N1—Fe1	124.13 (16)	C26—C25—C23	121.8 (2)
C5—N1—Fe1	119.29 (16)	C26—C25—H25	119.1
C19—N4—C18	116.9 (2)	C23—C25—H25	119.1
C15—N3—C11	116.3 (2)	C36—C34—C32	119.2 (2)
C15—N3—Fe1	116.29 (15)	C36—C34—C35	117.16 (19)
C11—N3—Fe1	126.34 (16)	C32—C34—C35	123.08 (19)
C9—C10—C6	119.2 (2)	C25—C23—C22	118.5 (2)
C9—C10—H10	120.4	C25—C23—C24	118.8 (2)
C6—C10—H10	120.4	C22—C23—C24	122.7 (2)
N1—C5—C4	124.2 (2)	O6—C33—O5	124.2 (2)
N1—C5—H5A	117.9	O6—C33—C32	123.6 (2)
C4—C5—H5A	117.9	O5—C33—C32	112.2 (2)
C1—C2—C3	120.4 (2)	C22—C28—C27	122.2 (2)
C1—C2—H2	119.8	C22—C28—H28	118.9
C3—C2—H2	119.8	C27—C28—H28	118.9
C7—C6—C10	116.5 (2)	C31—C30—C29	120.0 (2)
C7—C6—C3	121.9 (2)	C31—C30—H30	120.0
C10—C6—C3	121.6 (2)	C29—C30—H30	120.0
N1—C1—C2	123.4 (2)	O4—C24—O3	124.5 (2)
N1—C1—H1	118.3	O4—C24—C23	122.5 (2)
C2—C1—H1	118.3	O3—C24—C23	113.0 (2)
C5—C4—C3	119.6 (2)	C31—C32—C34	118.6 (2)
C5—C4—H4	120.2	C31—C32—C33	118.0 (2)
C3—C4—H4	120.2	C34—C32—C33	123.4 (2)
N2—C9—C10	124.1 (2)	C30—C31—C32	121.6 (2)
N2—C9—H9	117.9	C30—C31—H31	119.2
C10—C9—H9	117.9	C32—C31—H31	119.2
C6—C7—C8	119.9 (3)	C21—O2—Fe1	93.32 (13)
C6—C7—H7	120.0	C21—O1—Fe1	85.40 (14)
C8—C7—H7	120.0	O7—C35—O8	124.6 (2)
N2—C8—C7	123.5 (3)	O7—C35—C34	117.7 (2)
N2—C8—H8	118.3	O8—C35—C34	117.57 (19)
C7—C8—H8	118.3	C35—O7—Fe1^iii^	144.35 (15)
C11—C12—C13	119.1 (2)	C35—O8—Fe1^i^	128.26 (15)
C11—C12—H12	120.4		

Symmetry codes: (i) −*x*+1, −*y*+2, −*z*+1; (ii) *x*, *y*, *z*+1; (iii) *x*, *y*, *z*−1.

### Hydrogen-bond geometry (Å, °)

**Table:**

*D*—H···*A*	*D*—H	H···*A*	*D*···*A*	*D*—H···*A*
O5—H5···N2^iv^	0.82	1.86	2.677 (2)	171
O3—H3···N4^v^	0.82	1.81	2.598 (3)	162

Symmetry codes: (iv) *x*−1, *y*, *z*−2; (v) *x*+1, *y*, *z*+1.
